# Effect of topography and weather on delivery of automatic electrical defibrillator by drone for out-of-hospital cardiac arrest

**DOI:** 10.1038/s41598-021-03648-3

**Published:** 2021-12-17

**Authors:** Dong Sun Choi, Ki Jeong Hong, Sang Do Shin, Chang-Gun Lee, Tae Han Kim, Youngeun Cho, Kyoung Jun Song, Young Sun Ro, Jeong Ho Park, Ki Hong Kim

**Affiliations:** 1 Department of Emergency Medicine, Uijeongbu Eulji Medical Center, Uijeongbu, Gyunggi-do Republic of Korea; 2 Laboratory of Emergency Medical Services, Seoul National University Hospital Biomedical Research Institute, Seoul, Republic of Korea; 3 Department of Emergency Medicine, Seoul National University College of Medicine, Seoul National University Hospital, 101, Daehak-ro, Jongno-gu, Seoul, 03080 Republic of Korea; 4 Department of Computer Science and Engineering, Seoul National University College of Engineering, Seoul, Republic of Korea; 5 Department of Emergency Medicine, Seoul National University Seoul Metropolitan Government Boramae Medical Center, Seoul, Republic of Korea

**Keywords:** Cardiology, Health care, Medical research, Engineering

## Abstract

Delivery of automatic electrical defibrillator (AED) by unmanned aerial vehicle (UAV) was suggested for out-of-hospital cardiac arrest (OHCA). The goal of this study is to assess the effect of topographic and weather conditions on call to AED attach time by UAV-AED. We included OHCA patients from 2013 to 2016 in Seoul, South Korea. We developed a UAV-AED flight simulator using topographic information of Seoul for Euclidean and topographic flight pathway including vertical flight to overcome high-rise structures. We used 4 kinds of UAV flight scenarios according to weather conditions or visibility. Primary outcome was emergency medical service (EMS) call to AED attach time. Secondary outcome was pre-arrival rate of UAV-AED before current EMS based AED delivery. Call to AED attach time in topographic pathway was 7.0 min in flight and control advanced UAV and 8.0 min in basic UAV model. Pre-arrival rate in Euclidean pathway was 38.0% and 16.3% for flight and control advanced UAV and basic UAV. Pre-arrival rate in the topographic pathway was 27.0% and 11.7%, respectively. UAV-AED topographic flight took longer call to AED attach time than Euclidean pathway. Pre-arrival rate of flight and control advanced UAV was decreased in topographic flight pathway compared to Euclidean pathway.

## Introduction

### Background

Out-of-hospital cardiac arrest (OHCA) is one of the leading causes of death worldwide^1–3^. Early defibrillation is one of the important links to improve the survival rate of OHCA patients^4–7^. Public access defibrillation (PAD) programs have been implemented to reduce time between cardiac arrest to the first defibrillation^4, 8, 9^. However, incidence of OHCA located nearby PAD is limited. Moreover, it is difficult for the bystander to find the nearest PAD and apply it to the victims rapidly.

To overcome the limitation of current PAD installation strategy, delivery of automatic electrical defibrillator (AED) to the OHCA scene by unmanned aerial vehicle (UAV) such as drone has been suggested. The delivery of AED using drones was proposed to improve AED application rate and reduce defibrillation time^10–13^.

### Importance

Previous studies on UAV delivering AED (UAV-AED) program showed several limitations on its implementation to real clinical practice. First, control of UAV flight to the scene could be limited by weather conditions like rain, snow, wind speed and temperature. Poor visibility during flight due to nighttime or short sight distance by fog could also affect flight permission of UAV. The UAV could not safely and rapidly deliver AED in extreme weather conditions or with poor visibility. Second, topographic conditions could also increase the UAV flight time to the scene and weaken the benefit of UAV-AED program. Previous simulation studies reported the effect of UAV-AED program based on the flight route of UAV by the Euclidean distance from drone installation site to the location of OHCA victims^10–13^. However, to fly across the high-rise buildings in real life, vertical movement should be added to horizontal movement regarding Euclidean distance.

### Goal of this investigation

In this study, we developed a virtual UAV-AED flight simulation using topographic information such as natural terrain and buildings in Seoul, South Korea. We also added the meteorological information into the simulation scenario to permit flight of UAV-AED into the scene safely in metropolitan city.

The goal of this study is to assess the effect of topography and weather on call to AED attach time by UAV-AED program for OHCA. The hypothesis of this study is that adding topographic and weather conditions on the UAV-AED simulation program would increase call to AED attach time, unlike prior studies which did not consider these conditions.

## Methods

### Study design

This study is a retrospective observation study using a computerized virtual simulator. We included all OHCA cases registered in Korea OHCA Registry (KOHCAR) from 2013 to 2016, transported by emergency medical services (EMS) across South Korea. The Korea Center for Disease Control and Prevention (KCDC) approved use of all data and the study was approved by the Institutional Review Board Seoul National University Hospital (Approval number: H-1103-153-357). All methods were carried out in accordance with relevant guidelines and regulations. The informed consent was waived by the Institutional Review Board Seoul National University Hospital.

### Data source

The KOHCAR is a nationwide database including all cardiac arrest patients transported by EMS ambulances operated by fire departments across South Korea since 2006. This database registers prehospital information written by emergency medical technicians of National Fire Agency. Trained medical record reviewers of KCDC visited the hospitals, in which the OHCA patients were transported by National Fire Agency. They collected in-hospital information and outcome of OHCA victims. Then, prehospital and in-hospital data of each case was merged using the Utstein guidelines^14, 15^.

To develop a UAV-AED flight simulation, we initially extracted data on topography and altitude of Seoul city from Google maps. We constructed a geographic information database including height of buildings by combining the data about altitudes of each terrain with the altitude of all facilities supported by Ministry of Government Administration and Home Affairs. We also added meteorological information including wind speed, precipitation, snowfall, temperature, visibility and weather phenomenon, which were hourly recorded on Korea Meteorological Administration database. These data were added for scenario models of limited UAV-AED flight due to extreme weather condition.

### Study setting

Seoul has a population of 9.7 million and covers a total surface area of 605.2 km^2^. Seoul is a metropolitan city with many high-rise buildings and mountains. The EMS system of Seoul is a two-tiered public service model with service level of EMT-intermediate. Seoul is divided into 25 districts. Each district has 3 to 8 fire stations with ambulance vehicles and there are 116 fire stations in total^16^. Each ambulance vehicle is usually staffed with 3 EMTs^2, 17^. One dispatch center covers all EMS calls across Seoul^18^. The PAD installation is mandatory on public health offices, ambulances, airport, train, and apartments with more than 500 households. Approximately 8,000 AEDs are installed in Seoul^19^.

### Study population

All OHCA cases in Seoul from January 1, 2013 to December 31, 2016 with age of ≥ 9 years at cardiac arrest recognition time were eligible for this study. Patients with OHCA occurrence in the ambulance during EMS transport was excluded. Moreover, we excluded pediatric OHCA cases aged under 8 years. This is because pediatric cases aged under 8 were recommended for dose attenuator usage to optimize defibrillation energy^20^. Cases with missing AED attach time or cardiac arrest recognition time were also excluded.

### Variables

We used the Utstein variables from KOHCAR database such as gender, age, witnessed status, location of event (private vs public vs unknown), bystander CPR, initial electrocardiogram (ECG) rhythm, and EMS defibrillation^21^. We collected the EMS time profiles including EMS call time, EMS arrival at the scene time, EMS departure from the scene time, EMS hospital arrival time, call to cardiac arrest recognition time and call to AED attach time.

The address recorded on KOHCAR was used as the place of cardiac arrest. Geo-coding for the place of cardiac arrest was performed using Google Maps APIs (Google, California, United States). Regarding the weather-related variables, we collected hourly wind speed, precipitation, snowfall, temperature, visibility and weather phenomenon. Daytime and nighttime of OHCA occurrence was divided by 6AM and 6PM.

### Development of UAV-AED flight simulation

#### UAV-AED station allocation

All of 116 fire stations in Seoul were used as the candidates for UAV-AED installation. Among the 116 stations, the optimal location for each number of stations increased by 5 (i.e. 5, 10, 15, etc.) was selected for simulation from 5 to 116 stations. A multicriteria evaluation was conducted for selecting optimal combinations of possible UAV-AED installed stations to reduce call to AED attach time. We generated an OHCA occurrence layer by heat-map analysis of the OHCA occurrence location from 2013 to 2016 in Seoul. Each OHCA occurrence location was analyzed with a heat-map using a radius of 300 m. (Appendix 1) The EMS call to scene arrival time was analyzed by inverse distance weight (IDW) interpolation with a power coefficient of 2 using IDW plug-in in qGIS 3.4. to obtain EMS-response time layer. (Appendix 2) The OHCA risk map was calculated by adding 1: 1 weighting of OHCA occurrence layer and EMS call to scene arrival time layer. GIS analysis was performed using qGIS 3.4. The OHCA risk map was constructed with a lattice with a resolution of 50 m × 50 m. For optimal number of UAV-AED stations, the estimated coverage of each UAV-AED station was obtained by allocating a 3 km circle for each station. The optimal location for each number of stations, increased by 5, was selected according to the location with maximum score of OHCA risk map, which was calculated using the genetic algorithm^22^.

#### UAV-AED flight simulation

**Figure 1:**
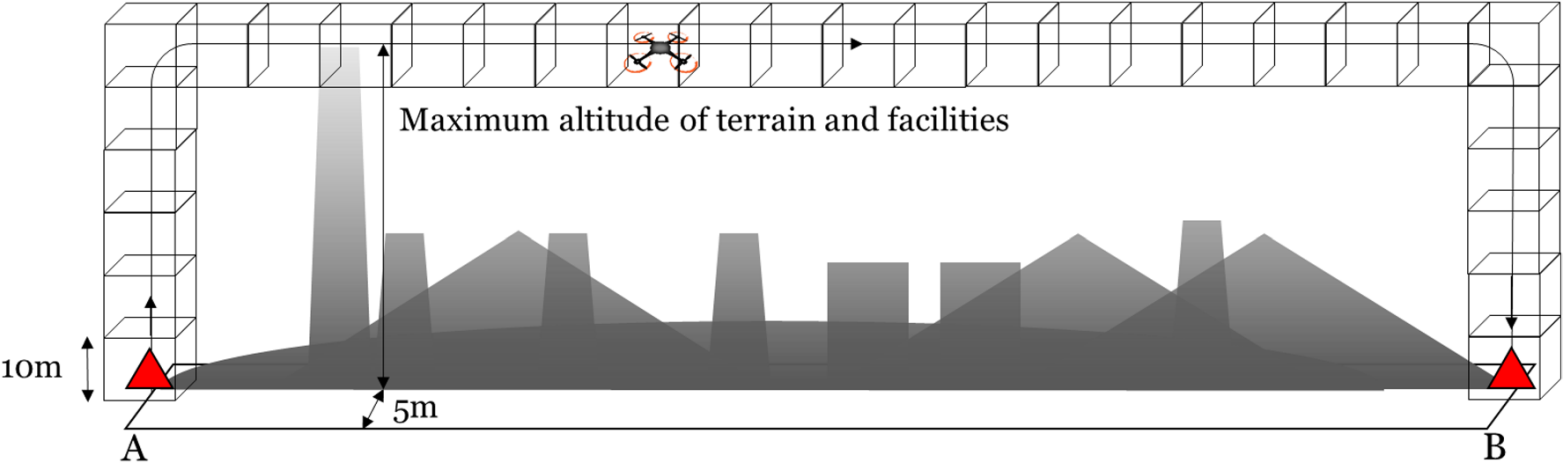
The topographic flight pathway used in the UAV-AED virtual flight simulator. (**A**) UAV-AED allocated station, (**B**) The site occurred out of hospital cardiac arrest.

UAV-AED flight simulation consists of environment information of Seoul and drone flight operation. Environmental information of Seoul was constructed by combining topographic information of natural terrain and facility information including location and height of high-rise buildings^23^. The UAV-AED topographic flight pathway was defined by 3 components. The first component was for the UAV-AED to take off vertically from the UAV-AED allocated station above the maximal altitude of natural terrain or high-rise buildings between UAV-AED station and OHCA site (Fig. 1). The second was horizontal flight to the OHCA site according to Euclidean distance. The final component was vertical landing of UAV-AED to the OHCA site. The entire flight pathway including take-off, horizontal flight and landing from UAV-AED station to OHCA site was divided by 3-dimensional virtual blocks of 10 m × 10 m × 10 m. The flight time of UAV-AED was defined as the sum of time required for passing all blocks in the flight pathway (Fig. 1). The block traversal time database is constructed with the measured flight times with varying entry speed, entry direction, escape speed, and escape direction. For example, a 10 km/h down-entry followed by a 10 km/h right-escape takes 3.25 s on average (Appendix 3). The flight time was computed by simulating the passage time of each block by HackflightSim^24^. The flight performance of UAV used for simulation was carried out based on performance of a Huesin Blueye 1 k model (Huins Inc., Gyunggi-do, South Korea) weighting 1.2 kg and moving up to 50 km/h^25^. The UAV flight simulation tests were performed using the dynamic simulator of drone transfer simulator on the reference webpage^26^.

#### UAV-AED simulation scenarios according to topographic and weather conditions

**Figure 2:**
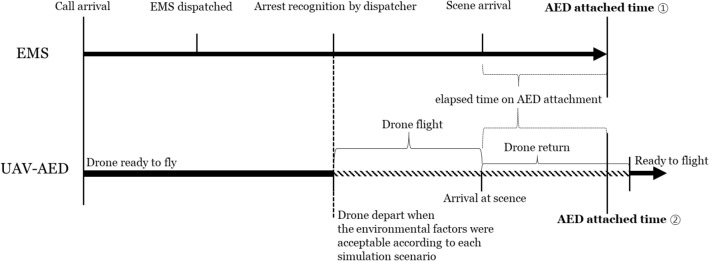
The timeline of call to AED attachment in EMS and UAV-AED simulation.

The detailed timeline of this simulation was shown in Fig. 2. In this study, it was assumed that the drone was dispatched when cardiac arrest was recognized, which was defined as the time at which the dispatcher-assisted CPR instruction was initiated. The drone was dispatched from the nearest drone station where there was a available drone that could be dispatched at that time. Based on meteorological information of Seoul during study period, we simulated 4 scenarios according to flight performance of the UAV regarding weather and visibility (Appendix 4). For each scenario, the availability of the drone is determined according to the meteorological conditions at the time the call was received. The first scenario was basic UAV model. In this model, flight of UAV-AED was restricted if EMS call for OHCA occurred during extreme weather conditions, which were defined as strong winds of 10 km/h or higher, rain, snow, and temperature below 0 °C. Also, if the call was made during nighttime or if the sight distance was less than 1 km, flight of UAV in the basic model was not permitted. The second model, control advanced UAV could fly regardless of time or limited visibility during flight. However, it was prohibited for use during extreme weather conditions. The third model, flight advanced UAV could fly in extreme weather conditions, but it could not fly in situations of poor visibility. Lastly, the flight and control advanced UAV model could fly whenever during the study period regardless of weather conditions or poor visibility. We simulated these 4 types of UAV model scenarios for 2 different flight pathways. The first flight pathway is the direct flight route through Euclidean distance from UAV-AED station to OHCA site. The second topographic flight pathway was generated by UAV-AED flight simulation developed in this study using topographic information.

### Outcome

Primary outcome was call to AED attach time. Call to AED attach time profiles by current EMS practice was measured by time profiles in the KOHCAR database; and time profiles by UAV-AED was measured by profiles derived from UAV-AED simulation. Secondary outcome was success rate of call to AED attach within 5 min or 10 min, and pre-arrival rate of UAV-AED before current EMS based AED delivery.

### Statistical analysis

The paired Wilcoxon rank sum test was used to compare the call to AED attach time between current practice and UAV-AED program. Call to AED attach success rate within 5 or 10 min before and after UAV-AED program implementation was compared using McNemar test. Call to AED attach time was compared according to the 4 drone flight simulation scenarios in both Euclidean distance pathway and topographic simulation pathway using the paired Wilcoxon rank-sum test. We used SAS 9.4.(NC, USA) for statistical analysis.

## Results

Total of 18,856 OHCA cases were registered in KOHCAR database during the study period. Among them, 194 patients with age of < 8 years, 1152 patients with OHCA occurrence during ambulance transport, 579 patients missing AED attach time record, and 335 patients missing OHCA recognition time record were excluded (Table 1).

**Table 1:** Demographic characteristics and weather related factors of study population.

Characteristics	N	%
Number	16,596	
Age, median (IQR)	69.4	(54.3–79.5)
**Gender**		
Male	10,569	63.7
Female	6027	36.3
Witnessed arrest	7133	43.0
Bystander AED apply	526	3.2
Bystander defibrillation	112	0.7
**EMS initial rhythm**		
Shockable	2488	15.0
Non-shockable	13,974	84.2
Unknown	134	0.8
EMS defibrillation	3535	21.3
Call to arrest recognition (min), median (IQR)	2.4	(1.6–3.4)
Call to EMS departure time (min), median (IQR)	1	(0–1)
EMS response time (min), median (IQR)	6	(4–7)
Scene arrival to AED attach time (min), median (IQR)	2	(1–4)
Call to AED attach time (min), median (IQR)	8	(6–11)
**Visibility related environmental factors**		
Night time	6749	40.7
Sight distance < 1 km	1780	10.7
**Extreme weather conditions**		
Temperature < 0 °C	2658	16.0
Rain	1585	9.6
Snow	884	5.3
Lightening	84	0.5
Wind speed > 10 m/s	1	0.0

*IQR* interquartile, *EMS* emergency medical service.

3754 OHCA cases (22.6%) occurred in public space and 7133 cases were witnessed arrest (43.0%). AED attachment by bystander occurred in 526 patients (3.2%) and 112 patients (0.7%) received defibrillation. Initial shockable rhythm at EMS scene arrival was observed in 2488 cases (15.0%) and defibrillation by EMS provider was done in 3535 cases (21.3%). The median EMS response time was 6.0 min (IQR 4.0–7.0). Median call to AED attach time by EMS was 8.0 min (IQR 6.0–11.0).

The weather and visibility related factors during study period are described in Table 2. Total of 6749 (40.7%) OHCA cases occurred at night and 1780 (10.7%) cases had sight distance of less than 1 km. The number of OHCA calls during extreme weather conditions was 2658 (16.0%) cases at temperature of < 0 °C, 1585 (9.6%) during rain, 884 (5.3%) during snow, 84 (0.5%) during lightning, and 1 case during wind speed higher than 10 m/s.

**Table 2:** Comparison of AED delivery related outcomes between current EMS situation and UAV-AED program based on UAV-AED topographic flight simulation.

AED delivery related outcomes	Current EMS situation		UAV-AED program according to UAV simulation scenarios							
			Flight and control advanced UAV		Flight advanced UAV		Control advanced UAV		Basic UAV	
	N	%	N	%	N	%	N	%	N	%
Total	16,596									
number of UAV-AED dispatched			7489	45.1	4340	26.2	5481	33.0	3199	19.3
UAV-AED flight time (min), median (IQR)			2.6	2.1–3.2	2.6	2.1–3.2	2.6	2.1–3.2	2.6	2.1–3.2
Call to AED attach time at the scene (min), median (IQR)	8.0	6.0–11.0	7.0^a^	5.0–10.0	7.6^a^	5.7–10.0	7.0^a^	5.3–10.0	8.0^a^	6.0–10.0
Success rate of call to AED attach within 5 min	3402	20.5	4183^b^	25.2	3859^b^	23.3	3974^b^	23.9	3741^b^	22.5
Success rate of call to AED attach within 10 min	12,401	74.7	13,149^b^	79.2	12,838^b^	77.4	12,984^b^	78.2	12,742^b^	76.8
Pre-arrival rate of UAV-AED before current EMS based AED delivery			4477	27.0	2590	15.6	3320	20.0	1,940	11.7

*UAV-AED* unmanned aerial vehicle delivering automatic electrical defibrillator, *IQR* interquartile.

^a^Paired Wilcoxon rank-sum test was significant (*p* < 0.05) compared to current EMS situation group.

^b^McNemar test was significant (*p* < 0.05) compared to current EMS situation group.

**Figure 3:**
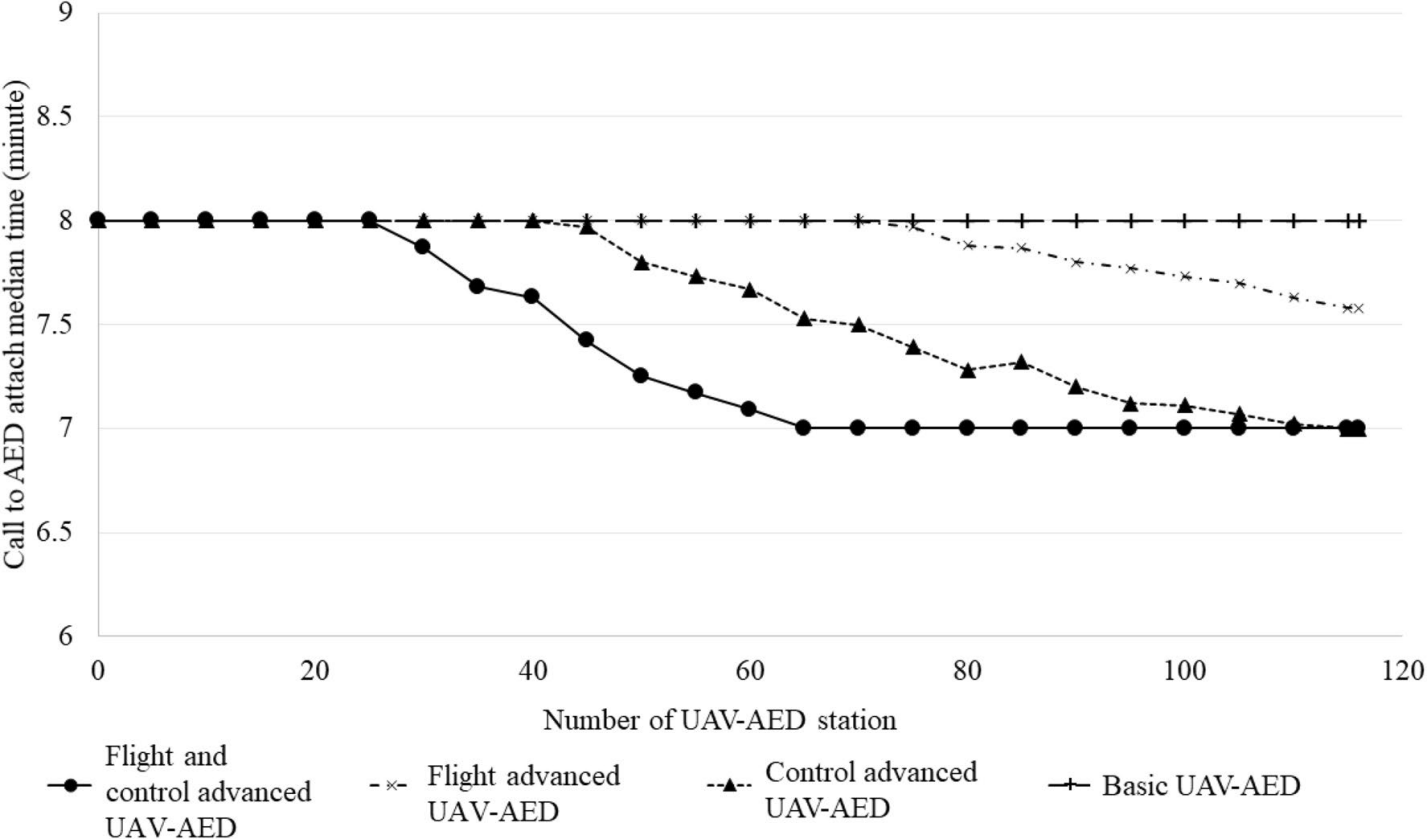
Call to AED attach time according to flight simulation scenarios and number of UAV-AED installed stations based on UAV-AED topographic flight pathway.

Call to AED attach time according to flight simulation scenario and number of UAV-AED stations in the UAV-AED topographic flight simulation are shown in Fig. 3. Call to AED attach time did not decrease in basic UAV model despite all 116 drone stations were available. Flight advanced model reduced call to AED attach time after operating more than 70 drone stations. Control advanced UAV decreased call to attach time from 45 stations. Call to attach time decreased in flight and control advanced UAV model with operation of 30 stations. Median flight time of UAV-AED in the topographic flight simulation was 2.6 min (Table 2). Median call to attach time was 7.0 min in flight and control advanced UAV and control advanced UAV models. Flight advanced UAV showed 7.6 min of call to AED attach time, whereas basic UAV showed 8.0 min. Success rate of call to AED attach time within 5 min was 25.0%, 23.9%, 23.3% and 22.5% in each UAV model, respectively. Pre-arrival rate of UAV-AED before current EMS based AED delivery was 27.0% in flight and control advanced UAV model and 11.7% in basic UAV model.

**Figure 4:**
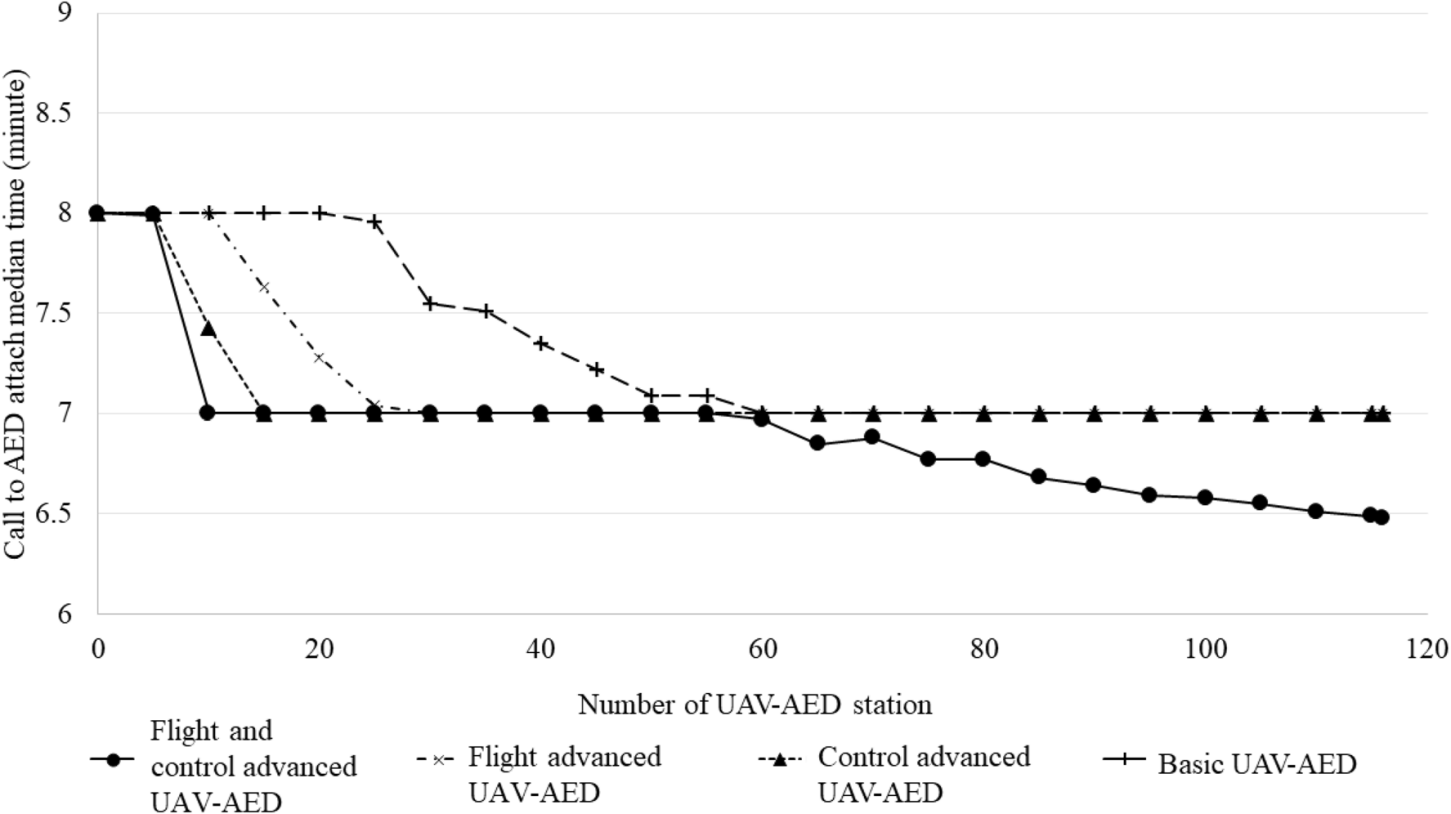
Call to AED attach time according to flight simulation scenarios and number of UAV-AED installed stations based on Euclidean flight pathway.

In Euclidean flight pathway, all UAV models showed reduced call to attach time compared to current EMS call to AED attach time. Moreover, decrease of call to AED attach time was observed even with lower number of operating drone stations in Euclidean pathway compared to topographic flight simulation (Fig. 4). Median flight time of UAV-AED in Euclidean flight pathway was 1.0 min (Table 3). The median call to attach time was 6.5 min in flight and control advanced UAV and 7.0 min in the other 3 UAV models. Success rate of call to AED attach time within 5 min was 34.8%, 31.3%, 28.8%, 26.7% in each UAV model. Pre-arrival rate of UAV-AED before current EMS based AED delivery was 38.0% in flight and control advanced UAV model and 16.3% in basic UAV model.

**Table 3:** Comparison of AED delivery related outcomes between current EMS situation and UAV-AED program based on Euclidean distance flight simulation.

AED delivery related outcomes	Current EMS situation		UAV-AED program according to UAV simulation scenarios							
			Flight and control advanced UAV		Flight advanced UAV		Control advanced UAV		Basic UAV	
	N	%	N	%	N	%	N	%	N	%
Total	16,596									
Number of UAV-AED dispatched			7489	45.1	4340	26.2	5481	33.0	3199	19.3
UAV-AED flight time(min), median (IQR)			1.0	0.7–1.3	1.0	0.7–1.3	1.0	0.7–1.3	1.0	0.7–1.3
Call to AED attach time at the scene (min), median (IQR)	8.0	6.0–11.0	6.5^a^	4.4–9.3	7.0^a^	5.0–10.0	7.0^a^	5.0–10.0	7.0^a^	5.0–10.0
Success rate of call to AED attach within 5 min	3402	20.5	5773^b^	34.8	4786^b^	28.8	5171^b^	31.2	4439^b^	26.7
Success rate of call to AED attach within 10 min	12,401	74.7	13,346^b^	80.4	12,959^b^	78.1	13,132^b^	79.1	12,830^b^	77.3
Pre-arrival rate of UAV-AED before current EMS based AED delivery			6304	38.0	3631	21.9	4631	27.9	2697	16.3

*UAV-AED* Unmanned aerial vehicle delivering automatic electrical defibrillator, *IQR* interquartile.

^a^Paired Wilcoxon rank-sum test was significant (*p* < 0.05) compared to current EMS situation group.

^b^McNemar test was significant (*p* < 0.05) compared to current EMS situation group.

## Discussion

In this study, we assessed the effects of topography and weather-related factors on flight performance of UAV regarding call to AED attach time by UAV-AED delivery program for OHCA cases in metropolitan city with high-rise buildings and mountains. This study added 3-dimensional topographic information and meteorological information of Seoul into the computerized virtual flight simulator. We analyzed the simulated results using the nationwide database of OHCA by merging prehospital and in-hospital clinical information across South Korea.

The median flight time was 1.0 min by Euclidean pathway flight simulation method used in previous studies. However, the median flight time increased to 2.6 min in the topographic pathway flight simulation. Longer flight distance was required to overcome flight barriers like mountains or high-rise buildings regarding Euclidean distance. Median call to attach time was 7.0 min in basic UAV model in Euclidean pathway. In topographic pathway, call to attach time was 8 min in basic UAV. Success rate of call to AED attach time within 5 min of flight and control advanced UAV was 34.8% in Euclidean pathway and 25.0% in topographic pathway. Therefore, gain in reduction of AED delivery time of UAV-AED program could be weakened in real-life environments with high-rise buildings, extreme weathers and poor visibility in metropolitan cities like Seoul.

An example of OHCA case applying the UAV-AED simulator results developed in this study is as follows. In the example case, the call time of OHCA case was 07:08. At that time, the temperature was 6.3 °C. The wind speed was 2.8 m/s and the sight distance was 1300 m. Other weather condition was clear. There was not poor visibility and extreme weather condition. Ground EMS ambulance was dispatched from the fire station which was 1068 m away from the OHCA site. It took 1 min to dispatch ground EMS ambulance and 3.05 min to recognize cardiac arrest. It took 7 min to arrive at the scene after dispatch and 2 min to attach the AED. The call to AED attach time by ground EMS was 10 min. In UAV-AED topographic flight simulation, the drone was dispatched at 3.05 min after EMS call from the nearest fire station 1,068 m away from the OHCA site. The height difference between the drone station and the OHCA site was 5 m, and the height of the tallest building in the route was 33 m. The flight time of the drone calculated by the topographic simulator was 3.17 min and it was assumed that it took 2 min to attach the AED after arrival at the site. The call to AED attach time by topographic AED-UAV simulator was 8.22 min. In the Euclidean distance simulation, the drone flight time was 1.28 min. The call to AED attach time was 6.33 min. During EMS activation to the OHCA site, ground ambulance could be dispatched immediately after address identification. But UAV-AED could be dispatched after cardiac arrest recognition. The advantage of rapid delivery of AED by UAV flight could be offset by adding cardiac arrest recognition time. And the presence of high-rise building like this case could affect the AED attach time. Compared to straight distance flight, the flight time of UAV-AED was increased by the simulator that reflects topographic information.

The effect of UAV-AED program on reducing call to AED attach time in this study was lower than the results of previous studies^10–13^. First, operation of UAV was limited by environment with poor visibility during flight. In situations of poor visibility, current UAV operated by remote control could not guarantee safe delivery of AED to the scene without collision with housing buildings or laypersons. Second, flight performance of current UAV was restricted under extreme weather conditions like raining or high wind speed. Third, traffic environment and EMS resources of metropolitan city showed shorter delivery time of AED to the scene by ambulance vehicle compared to rural areas. In Seoul, median call to AED attach time of current EMS was 8 min in this study. These results suggest that the effect of the UAV-AED shown in previous studies may have been overestimated when considering the environmental and geographical effects in an urban area where the emergency medical system is sufficiently organized.

UAV-AED was also advantageous in UAV-AED arrival time before current EMS based AED delivery in this study. However, optimized UAV-AED installation in each community was required to maximize the benefit of UAV-AED. Floating commercial drones with limited flight operation at night or in bad weather did not reduce call to AED attach time despite all EMS stations in Seoul were used for UAV-AED installation. Drones with augmented visibility or flight performance could overcome this restriction. Flight of UAV in metropolitan city by remote control was another limitation when finding the best route in metropolitan city with high-rise buildings. Designation of the flight pathway for UAV-AED based on 3-dimensional topographic information should be preceded for effective implementation of the UAV-AED program in metropolitan city.

There are some limitations to this study. First, this study was a simulation study using a computerized virtual flight simulator. Clinical trial of UAV-AED is required to assess clinical outcome or other operational factors affecting UAV-AED flight. Second, we used flight scenarios with flight speed of 50 km per hour and limited UAV flight operation Improvement of UAV flight performance may affect the result. Third, there is a limitation of generalizability. The goal of this study was to assess the effect of UAV-AED in metropolitan city like Seoul, South Korea. Difference of surface area, density or height of high-rise buildings, natural terrain and weather can affect the result.

In conclusion, effect of topography and weather condition took longer call to AED attach time in basic UAV than flight and control advanced UAV. And longer call to AED attach time was observed comparing with Euclidean pathway simulation. Pre-arrival rate of flight and control advanced UAV was decreased in topographic flight pathway compared to Euclidean pathway.

## Supplementary Information

Supplementary Information 1.
