# Supplementary material for: Effect of topography and weather on delivery of automatic electrical defibrillator by drone for out-of-hospital cardiac arrest
Source: Sci Rep. 2021 Dec 17;11:24195. doi: 10.1038/s41598-021-03648-3 (PMC8683495; doi:10.1038/s41598-021-03648-3)
Supplement: Supplementary file 1 — Supplementary Information 1. [file 41598_2021_3648_MOESM1_ESM.docx]

Appendix 1. The OHCA occurrence layer by heat-map analysis of OHCA occurrence location from 2013 to 2016 in Seoul. Each cardiac arrest event site was analyzed with a heat-map with a radius of 300 m. Figure created with QGIS. 3.4. Quantum GIS Geographic Information System. Open Source Geospatial Foundation Project. <http://www.qgis.org/en/site/>.


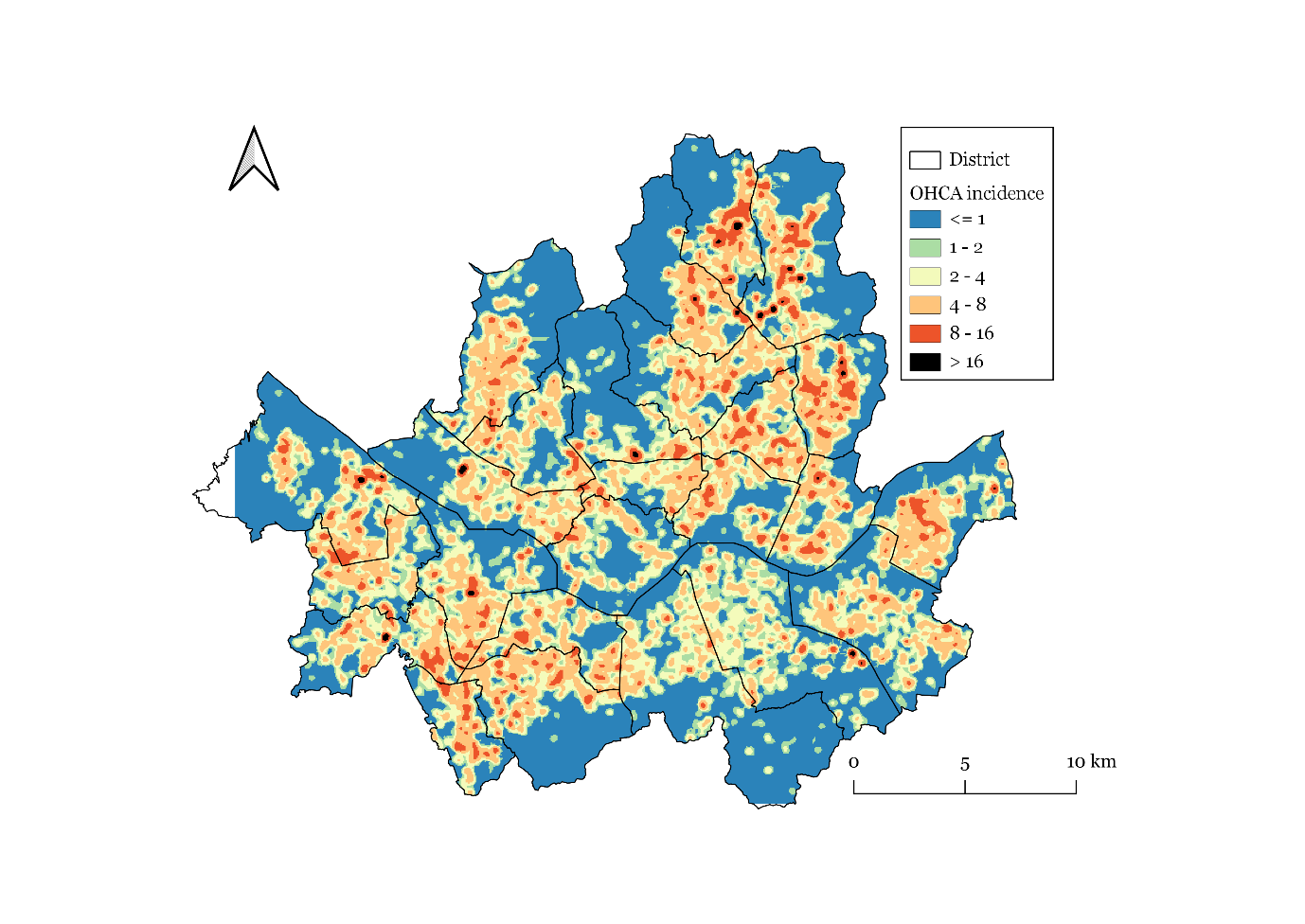


Appendix 2. The layer of EMS call to scene arrival time from 2013 to 2016 in Seoul after inverse distance weight (IDW) interpolation and location of 116 fire stations. Figure created with QGIS. 3.4. Quantum GIS Geographic Information System. Open Source Geospatial Foundation Project. <http://www.qgis.org/en/site/>
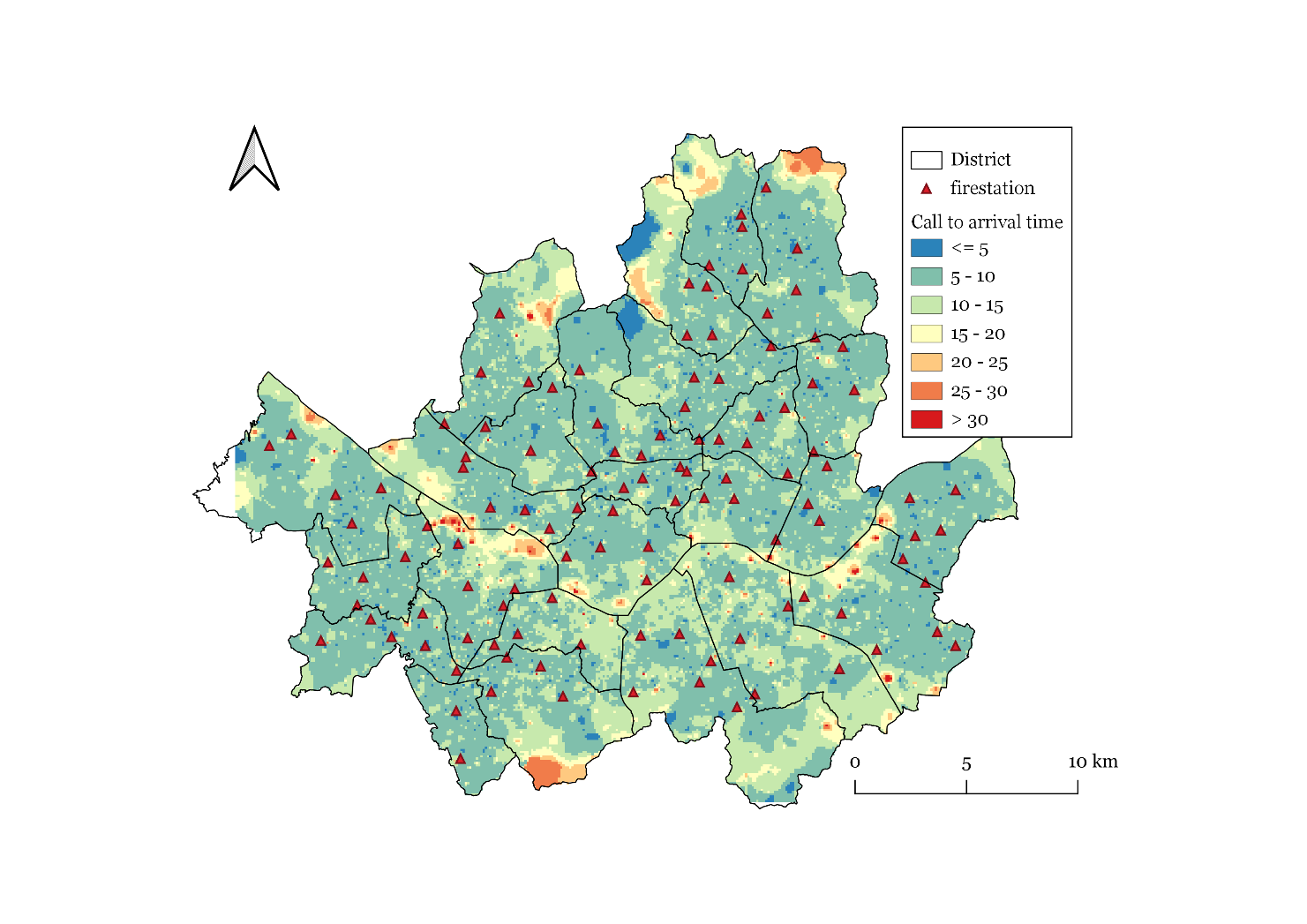


Appendix 3. Example of UAV elapsed time database according to the entery speed and direction and the escape speed and direction.


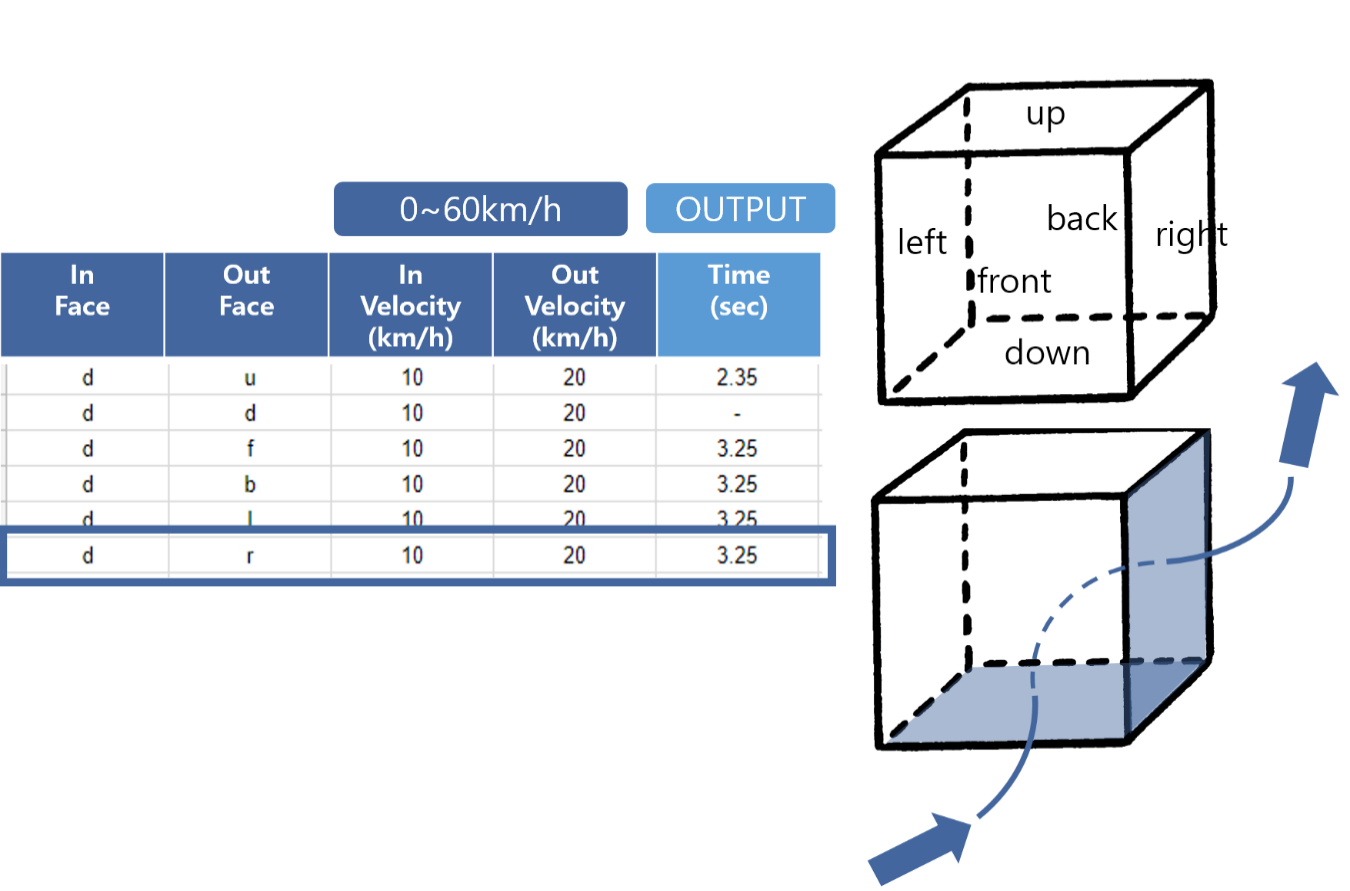


Appendix 4. Speculation of each UAV type according to environmental factors

| Environmental factors | Basic UAV | Control advanced UAV | Flight advanced UAV | Flight and control advanced UAV |
| --- | --- | --- | --- | --- |
| Poor visibility related environments (Night time,  Sight distance < 1km) | Unavailable | Available | Unavailable | Available |
| Extreme weather conditions  (Temp <0℃, Rain, Snow, Lightening, Wind speed > 10m/s ) | Unavailable | Unavailable | Available | Available |
| Required specification | Maximum speed : 50km/s  Maximum operation distance : 10km  GPS based Autopilot system | Basic UAV  + Anti-Collison light,  Augmented visual system such as Infra-red (IR) camera | Basic UAV  +  waterproof  anti-lightning  wind-resistance  cold-resistance | Basic UAV  +  Anti-Collison light,  Augmented visual system such as Infra-red (IR) camera  waterproof  anti-lightning  wind-resistance  cold-resistance |
